# Performance Enhancement of CdS/CdSe Quantum Dot-Sensitized Solar Cells with (001)-Oriented Anatase TiO_2_ Nanosheets Photoanode

**DOI:** 10.1186/s11671-018-2842-5

**Published:** 2019-01-11

**Authors:** Kuo-Yen Huang, Yi-Hsiang Luo, Hsin-Ming Cheng, Jau Tang, Jin-Hua Huang

**Affiliations:** 1 Department of Materials Science and Engineering, National Tsing Hua University, Hsinchu, 300 Taiwan; 2 Research Center for Applied Sciences (RCAS), Academia Sinica, Taipei, 115 Taiwan

**Keywords:** CdS/CdSe QDSSCs, TiO_2_ nanosheets, Photoanode

## Abstract

CdS/CdSe quantum dot-sensitized solar cells (QDSSCs) were fabricated on two types of TiO_2_ photoanodes, namely nanosheets (NSs) and nanoparticles. The TiO_2_ NSs with high (001)-exposed facets were prepared via a hydrothermal method, while the TiO_2_ nanoparticles used the commercial Degussa P-25. It was found that the pore size, specific surface area, porosity, and electron transport properties of TiO_2_ NSs were generally superior to those of P-25. As a result, the TiO_2_ NS-based CdS/CdSe QDSSC has exhibited a power conversion efficiency of 4.42%, which corresponds to a 54% improvement in comparison with the P-25-based reference cell. This study provides an effective photoanode design using nanostructure approach to improve the performance of TiO_2_-based QDSSCs.

**Electronic supplementary material:**

The online version of this article (10.1186/s11671-018-2842-5) contains supplementary material, which is available to authorized users.

## Background

In recent years, quantum dot-sensitized solar cells (QDSSCs) have attracted considerable attention as promising alternatives to dye-sensitized solar cells (DSSCs). The specific advantages of quantum dots (QDs) over organic dyes and Ru-based dyes include larger extinction coefficient, tunable energy bandgap by controlling the dot size and chemical composition, higher photonic and chemical stability, and possibility for multiple exciton generation and hot carrier transfer [1–4]. Theoretically, QDSSCs can enhance the light-to-electricity conversion efficiency beyond the Shockley-Queisser limit of 32% [5].

The photoelectric conversion scheme of QDSSCs is similar to that of DSSCs but using inorganic nanocrystals instead of organic dyes as light absorbers. Generally, QDSSCs consist of a QD-coated metal oxide as the photoanode, polysulfide complex (*S*^2−^/*S*_*x*_^2−^) as the liquid redox electrolyte, and Pt metal as the counter electrode. Many kinds of narrow bandgap semiconductor QDs, such as CdS, CdSe, CdTe, and PbS, have been utilized as light absorbers in the visible light regime [6–10]. To extend the light absorption range and facilitate the carrier injection in QDSSCs, the QDs with appropriate energy level matching, such as CuInS_2_/CdS [11, 12], CdTe/CdSe [13], and CdS/CdSe [14–21], have been combined to form core/shell structure QD co-sensitizers. Among them, the CdS/CdSe core/shell structure QDs have been widely studied due to their relative stability and simple synthesis, and the resulting cells generally exhibited power conversion efficiencies of < 5%. At present, the reported best-performing QDSSCs still exhibit moderate power conversion efficiencies of 6–8% [10, 13, 22, 23] due to serious charge recombination and low QD coverage on the photoanodes. To further improve the performances of QDSSCs, the present strategy has focused on using the mesoporous metal oxides as photoanode materials to enhance the electron transport, light harvesting, and QDs loading.

In both QDSSCs and DSSCs, TiO_2_ has been a preferred porous photoanode material because of its high efficiency, low cost, and excellent chemical stability [24]. It has been well known that the performance of TiO_2_-based photovoltaics is highly dependent on the morphology and crystal structure of TiO_2_, and the available anatase TiO_2_ nanoparticles (NPs) are mostly dominated by the thermodynamically stable (101) facets [25]. However, theoretical and experimental studies have demonstrated that the (001) facets are much more active than the thermodynamically stable (101) surfaces [26], which are favorable for dye or QD absorption and help to retard charge recombination [27–29]. Additionally, the band edge of the (001) facets has been confirmed to be lower than that of the (101) facets, which is advantageous for voltage enhancement [30].

Various TiO_2_ nanostructures with high (001)-exposed facets, including nanosheets (NSs), hollow spheres, and nanotubes [31–34], have been used in the DSSCs system. In particular, the anatase TiO_2_ NSs with a high percentage of (001)-exposed facets have been proven to exhibit unique surface structure characteristics which potentially lead to performance enhancements in water splitting, photocatalysis, and lithium-ion batteries [31, 35, 36]. However, to the best of our knowledge, there are much fewer reports on the use of the novel (001) facet-tailed TiO_2_ nanosheet structure in the QDSSCs system [28]. In this work, we present a comparative study on the photovoltaic performances of the TiO_2_ NS- and NP-based CdSe/CdS QDSSCs. The TiO_2_ NSs with high (001)-exposed facets were prepared via a hydrothermal method [37], while the TiO_2_ NPs used the commercial Degussa P-25. We found that the pore size, specific surface area, and porosity of TiO_2_ NSs were generally superior to those of P-25. The resulting TiO_2_ NS-based CdSe/CdS QDSSC exhibited an energy conversion efficiency of 4.42%, which is significantly enhanced by up to 54% as compared with the P-25-based reference cell under similar fabrication conditions.

## Methods

### Preparation of Various TiO_2_ Photoanodes

The anatase TiO_2_ NSs with high (001)-exposed facets were synthesized via a hydrothermal method [37]. Briefly, 2.4 ml hydrofluoric acid (Aldrich, 48 wt%) was first added dropwise into 30 ml titanium butoxide (Ti(OBu)_4_, Aldrich, > 97%), and the mixture was sealed into a dried Teflon-lined stainless steel autoclave. The synthesis process was then conducted at 180 °C for 16 h in an electric oven. The resulting TiO_2_ NS precipitates were collected by centrifugation and washed with deionized water and ethanol several times. Two kinds of screen-printable pastes, the TiO_2_ NSs and commercial P-25, were prepared by mixing 6 g of TiO_2_ NSs (or P-25 powder), 20 ml terpineol, and 30 ml 10 wt% ethyl cellulose (EC) in a round-bottomed rotovap flask. After sonicating and concentrating, the resulting 13 wt% homogenous pastes was coated on the fluorine-doped tin oxide (FTO) glass substrates (10 ohms per square, 2.2 mm thickness) by screen printing. Finally, the screen-printed TiO_2_ NSs and P-25 photoanodes were annealed at 500 °C for 1 h in air to allow good electrical conduction.

### Deposition and Sensitization of CdS/CdSe QDs

The deposition methods of QDs on metal oxides in QDSSCs can be classified into two types: (1) in situ growth via the successive ion-layer absorption and reaction (SILAR) process for CdS QDs and together with the chemical bath deposition (CBD) or chemical vapor deposition process for CdSe QDs; and (2) absorption of preprepared QD colloids via modified ligands. Although the latter method is easier to control the QD size and surface modification, the in situ growth associated with direct contact on the metal oxide method has lower fabrication cost [17]. In this work, the two distinct photoanodes, TiO_2_ NSs, and P-25, were also in situ sensitized with CdS and CdSe QDs using the SILAR and CBD processes, respectively. For the deposition of CdS QDs, two separate precursor solutions were prepared: 20 mM CdCl_2_ and 20 mM Na_2_S were dissolved in a mixture of methanol and deionized water (1:1, *v*/v) as cation and anion sources, respectively. Both the TiO_2_ NSs and P-25 photoanodes were first dipped into the Cd^2+^ precursor solution for 1 min, and then dipped into the S^2−^ precursor solution for 1 min. Before each immersion, the photoanodes were rinsed with methanol and then dried with N_2_ flow. These procedures were repeated several cycles to form a suitable CdS QD layer. For the subsequent deposition of CdSe QDs onto the CdS QDs, the TiO_2_/CdS photoanodes were dipped into an aqueous solution consisting of 2.5 mM Cd(CH_3_COO)_2_, 2.5 mM Na_2_SeSO_3_ and 75 mM NH_4_OH. The deposition process was maintained at 70 °C for 1 h. The loading of the CdSe QDs was controlled by adjusting the number of reaction cycles.

### Assembly and Characterization of QDSSCs

The various TiO_2_ based CdS/CdSe QDSSCs were assembled in a conventional sandwich structure. The platinum-coated FTO glass and CdS/CdSe QDs sensitized TiO_2_ photoanodes were sealed together, separating with a 25 μm hot-melting polymer spacer (DuPont Surlyn). The polysulfide electrolyte, which consisted of 0.2 M Na_2_S, 0.2 M S, and 0.02 M KCl in aqueous solution, was injected into the space between the electrodes. The active area of all QDSSCs was ~ 0.16 cm^2^ (~ 0.4 cm × 0.4 cm).

All CdS/CdSe QDSSCs were characterized using field emission scanning microscopy (FE-SEM, JEOL JSM-6500F), transmission electron microscopy (TEM, JEOL JEM-3000F and Hitachi HT7700), and glancing incident X-ray diffraction (GIXRD, PANalytical X’Pert PRO MPD). The loadings of QDs on the various TiO_2_ photoanodes were estimated by an inductively coupled plasma mass spectrometer (ICP-MS, Agilent 7500ce). The current-voltage characteristics and electrochemical impedance spectroscopy (EIS) measurements of the photovoltaic cells were performed under simulated one-sun illumination (100 mW/cm^2^, AM 1.5 G). The incident photon converted to current efficiency (IPCE) was measured by employing a 150-W XQ lamp with a monochromator under the DC mode. The optical absorbance was carried out with a UV-VIS spectrophotometer (Jasco V-670) with a tungsten halogen lamp.

## Results and Discussion

**Fig. 1:**
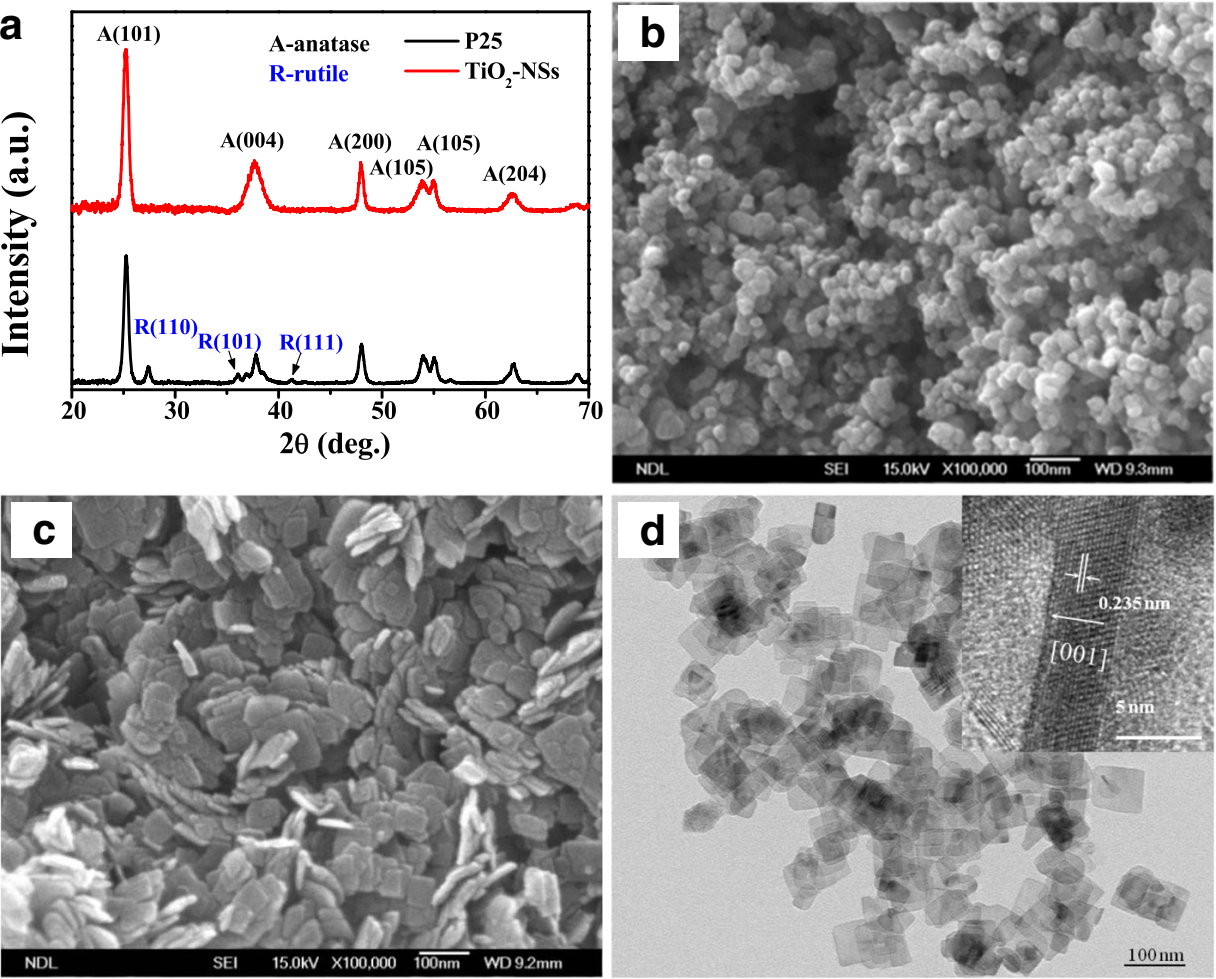
**a** XRD patterns of bare TiO_2_ NSs and P-25. **b**, **c** SEM images of bare TiO_2_ NSs and P-25, respectively. **d** TEM and HRTEM (insert) images of bare TiO_2_ NSs

**Fig. 2:**
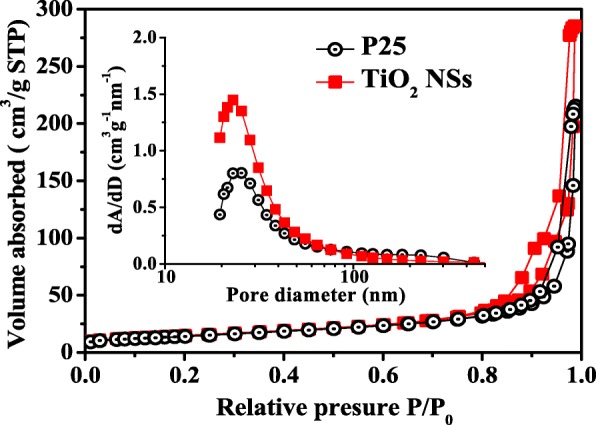
Nitrogen absorption-desorption isotherms and pore size distributions (insert) of bare TiO_2_ NSs and P-25

In this study, the anatase TiO_2_ NSs with high (001)-exposed facets were prepared as the photoanodes of QDSSCs via a hydrothermal method. Their performances were investigated, discussed, and compared with the commercial nanoporous Degussa P-25 photoanode. The crystal structure and composition of TiO_2_ NSs were characterized by X-ray diffractometry. As shown in Fig. 1a, all the identified peaks of TiO_2_ NSs can be indexed to a pure anatase TiO_2_ phase with a tetragonal structure and space group I4_1_/amd (JCPDS cards, No.71-1169), with no rutile phase being observed. The (004) and (200) reflection peaks represent the *c*- and *a*-axes, respectively. The enhanced sharp (200) peak indicates well-crystallized TiO_2_ NSs grown along the *a*-axis. A typical FE-SEM image of P-25 is shown in Fig. 1b. The FE-SEM and TEM images of TiO_2_ NSs are shown in Fig. 1c and d, respectively, which depict the well-defined sheet shape with an average side length of 50 nm and a thickness of 5 nm. The high-resolution TEM image (inset of Fig. 1d) shows the side view of a single TiO_2_ NS crystal. The lattice spacing of 0.235 nm can be directly observed, which corresponds to the (001) planes of the anatase TiO_2_ NSs. Analysis of the above results indicates ~ 70% of TiO_2_ NSs are comprised of the exposed (001) facets (see Additional file 1). In contrast, for the P-25, the percentage of exposed (001) facets is less than 10%, with over 90% dominated by the (101), (110), etc. facets. The specific surface area and pore size distribution of the TiO_2_ NSs and P-25 photoanodes were investigated using nitrogen absorption and desorption isotherms. As shown in Fig. 2, the isotherm of a TiO_2_ NS photoanode is identified as type IV based on the Brunauer-Deming-Deming-Teller (BDDT) classification [38]. The corresponding hysteresis loop at the high relative pressure (*P*/*P*_*o*_) range of 0.75–1 belongs to type H3, indicating the presence of slit-like mesopores and macropores. These types of porous structures render a relatively high surface area and large total pore volume. The BET-specific surface area was determined to be ~ 52.8 cm^2^ g^−1^, based on the Barrett-Joyner-Halenda (BJH) pore size distribution as shown in the inset of Fig. 2. Table 1 summarizes the detailed information about the surface structures of TiO_2_ NSs and P-25. The relatively larger crystal size, higher pore size, and bigger surface area of TiO_2_ NSs are beneficial to the absorption of the CdS/CdSe QDs.

**Table 1:** Comparison of the various physical properties of the TiO_2_ NSs and P-25 photoanodes

Sample	Phase^a^	*S*_BET_ (m^2^ g^−1^)	Pore size (nm)	Pore volume (cm^3^ g^−1^)	Porosity^b^ (%)
TiO_2_ NSs	A	52.83	28.42	0.44	63.25
P-25	A/R	50.89	24.30	0.33	57.04

^a^R and A denote the rutile and anatase structures, respectively

^b^Porosity = pore volume/(solid volume without pore + pore volume)

**Fig. 3:**
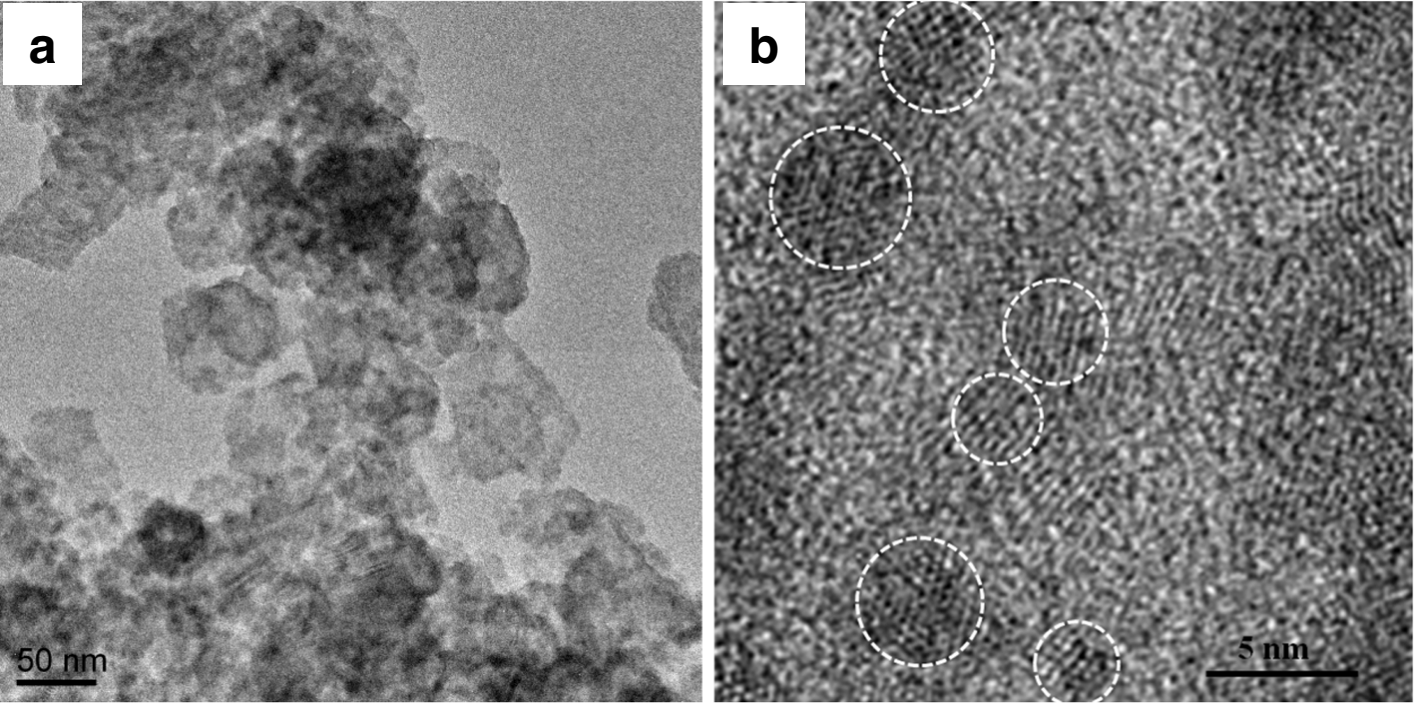
**a** TEM and (**b**) HRTEM images of CdS/CdSe-sensitized TiO_2_ NSs

The cascaded CdS/CdSe QDs have been extensively used as co-sensitizers for the QDSSCs because of their wide absorption range and good electron transfer dynamics [39]. In this work, the effects of the coating cycles of the SILAR (for CdS QDs) and CBD (for CdSe QDs) processes were first investigated, and the results revealed the optimum coating cycles of 8 and 2 for the CdS and CdSe QDs depositions, respectively. After deposition of the cascaded CdS/CdSe QDs by the two-step deposition process, the color of the TiO_2_ NS film turned from white to dark brown. Figure 3a displays a TEM image of the CdS/CdSe QD-sensitized TiO_2_ NSs scraped from the FTO glass substrate. It can be seen that dense CdSe nanocrystals have coated on the surface of TiO_2_ NSs without obvious aggregation. Furthermore, the lattice fringes of CdSe QDs can be clearly distinguished in the high-resolution TEM image in Fig. 3b, indicating the high crystallinity of CdSe QDs with a grain size ranging 4–6 nm.

**Fig. 4:**
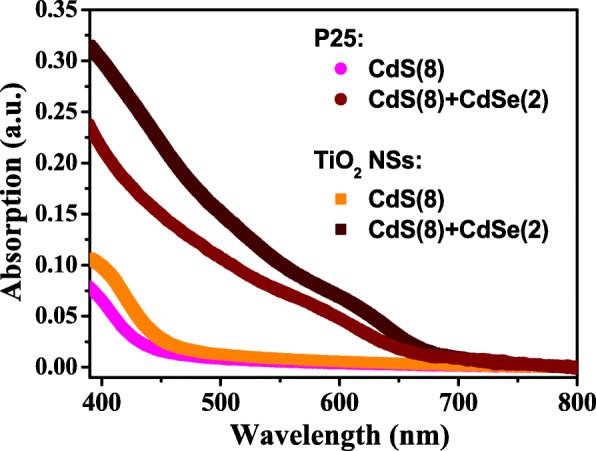
UV-VIS absorption spectra of ~ 3-μm-thick TiO_2_ NSs and P-25 sensitized by CdS and CdSe QDs. The number in parenthesis indicates the coating cycles of the SILAR (for CdS) and CBD (for CdSe) processes

**Fig. 5:**
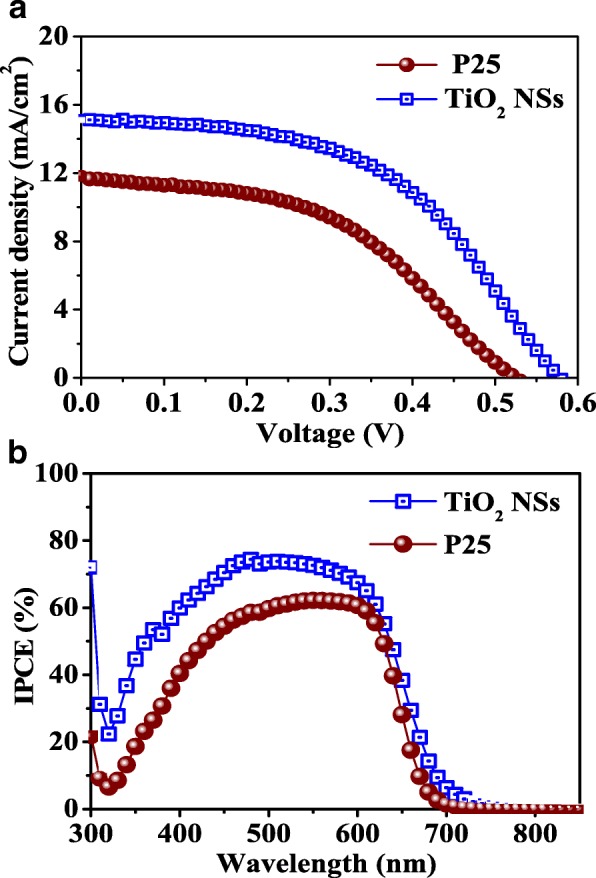
(**a**) J-V characteristics and (**b**) IPCE spectra of the TiO2 NSs and P-25-based QDSSCs

Figure 4 shows the UV-VIS absorption spectra of the CdS/CdSe QD-sensitized TiO_2_ NSs and P-25 electrodes prepared under similar deposition conditions. The excitonic absorption peaks usually observed in colloidal QDs were also detected here due to the broad range of size distribution of QDs fabricated by the SILAR and CBD processes. The corresponding bandgaps of the CdS and CdSe QDs can still be identified as 2.67 and 1.78 eV, respectively, by the absorption edges. Apparently, these values are larger than those of bulk CdS (2.25 eV) and CdSe (1.7 eV), indicating the particle sizes of the two nanocrystals are still within the scale of quantum confinement even after the sequentially chemical depositions. In the visible region, a higher absorption for the TiO_2_ NS electrode compared to the P-25 electrode is observed, implying that the loadings of CdS and CdSe QDs on the TiO_2_ NSs are higher than on the P-25. Furthermore, ICP-MS was used to obtain the qualitative QDs loading on the two different types of TiO_2_ photoanodes. By analyzing the results obtained from the BET and ICP-MS, the surface concentration of CdS QDs absorbed on the TiO_2_ NSs (5.44 × 10^−9^ mol cm^−2^) is found to be higher than that on the P-25 (4.59 × 10^−9^ mol cm^−2^). This verifies the reactive (001) facets of TiO_2_ NSs can afford more effective sites for attachment of CdS QDs, thereby providing higher absorbance of CdSe QDs on CdS QDs. As a result, the surface concentration of CdSe QDs on the TiO_2_ NS photoanode is also higher than that on the P-25 (4.57 × 10^−9^ mol cm^−2^ vs. 3.77 × 10^−9^ mol cm^−2^), which is consistent with the previously reported results [15]. The high (001)-exposed facets of TiO_2_ NSs apparently improve the surface concentration of CdSe/CdS co-sensitizers and thus increase the light harvesting of resulting QDSSCs. The photovoltaic performances of the TiO_2_ NS- and P-25-based CdSe/CdS QDSSCs were examined by characterizing their current-voltage behaviors under the simulated one-sun illumination (100 mW cm^−2^, AM 1.5 G). The TiO_2_ NSs and P-25 photoanodes under investigation are both ~ 10 μm thick. The *J*-*V* characteristics and incident photon-to-electron conversion efficiencies of the two QDSSCs are illustrated in Fig. 5, and their detailed photovoltaic parameters are tabulated in Table 2. It can be seen that the TiO_2_ NS-based QDSSC achieved a larger open-circuit voltage (*V*_oc_) of 0.58 V, a higher short-circuit current density (*J*_sc_) of 15.07 mA cm^−2^, and a better conversion efficiency (*η*) of 4.42% compared to the P-25-based QDSSC (*V*_oc_ = 0.52 V, *J*_sc_ = 11.75 mA cm^−2^, and *η* = 2.86%). The TiO_2_ NS-based QDSSC exhibits a 60-mV larger *V*_oc_ than the P-25-based cell. This enhancement of the open-circuit voltage in the TiO_2_ NS-based QDSSC can be attributed to the negative shift of the flat-band potential for the (001) facets [30]. On the other hand, it is well known that the *J*_sc_ is proportional to the amount of light absorbed on the metal oxide. Therefore, the larger *J*_sc_ in the TiO_2_ NS-based QDSSC is consistent with the result of ICP-MS, confirming the reactive anatase (001) facets favor the loading of quantum dots per unit area. Thus, the utilization of highly reactive TiO_2_ NSs as photoanodes can significantly improve the photocurrents of the TiO_2_-based photovoltaic devices. Moreover, the larger pore size of TiO_2_ NSs reduces the light scattering in the TiO_2_ NSs. This allows a longer distance that light can travel within the TiO_2_ NSs, thereby enhancing the electron absorption probability. As shown in Fig. 5b, the IPCE spectrum edge of the TiO_2_ NS-based QDSSC is located at 675 nm, which is slightly red-shifted when compared with the P-25-based QDSSC. In general, the IPCE value is determined by light harvesting efficiency, charge injection efficiency, and charge collection efficiency of the photoanode. The result is well matched with the UV-VIS absorption spectra, and the photocurrents integrated from the IPCE curves are in good agreement with the *J-V* measurements. Compared to the P-25-based QDSSC, the TiO_2_ NS-based QDSSC has higher IPCE values in the measuring range of 300–800 nm, with the maximum IPCE value of ~ 75%.

**Table 2:** Photovoltaic properties of the TiO_2_ NS- and P-25-based QDSSCs

Electrode	*V*_oc_ (V)	*J*_sc_ (mA/cm^2^)	FF	*η* (%)
TiO_2_ NSs	0.58	15.07	0.51	4.42
P-25	0.52	11.75	0.46	2.86

**Fig. 6:**
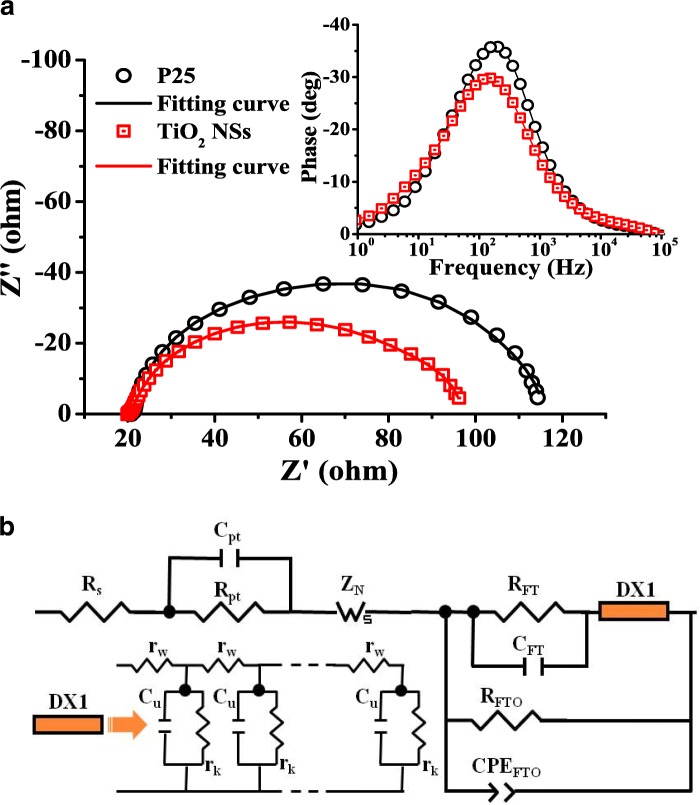
**a** Nyquist plots of the TiO_2_ NSs and P-25-based QDSSCs measured at *V*_oc_ under one-sun illumination. Inset: the corresponding phase Bode plots. **b** The equivalent circuit of QDSSCs, where *R*_s_ is the series resistance; *R*_pt_ and *C*_pt_ are the charge-transfer resistance and the interfacial capacitance at the Pt/electrolyte interface, respectively; *R*_FT_ and *C*_FT_ are the resistance and the interfacial capacitance at the FTO/TiO_2_ contact, respectively; *R*_FTO_ and CPE_FTO_ are the charge transfer resistance and the constant phase element of the electric double layer at the FTO/electrolyte interface, respectively

The highly reactive (001) surface of TiO_2_ NSs has been verified to offer a more effective surface area for QD absorption. Moreover, TiO_2_ NSs are expected to reduce the surface traps and recombination centers at the TiO_2_-NS/electrolyte interface for electron transport [37]. In order to acquire a better insight on the dynamics of the interfacial charge transfer and charge transport processes in the present QDSSCs, electrochemical impedance spectroscopy (EIS) measurements [40–42] were carried out. Figure 6a displays the Nyquist plots of both QDSSCs under one-sun illumination at the open-circuit voltage condition, in which the experimental data are represented with symbols and the solid line fitting curves were obtained by the Zview software using the QDSSC equivalent circuit as shown in Fig. 6b. The fitting parameters of electron transport are listed in Table 3, where *τ*_eff_ is the electron effective lifetime, *R*_*w*_ (= *r*_*w*_.*L*) is the electron transport resistance in the TiO_2_, *R*_*k*_ (= *r*_*k*_/*L*) is the charge transfer resistance related to recombination of electrons at the TiO_2_/electrolyte interface, *D*_*n*_ is the effective electron diffusion coefficient, *L*_*n*_ is the electron diffusion length in TiO_2_, and *L* (~ 10 μm) is the thickness of the electrodes. *D*_*n*_ is estimated according to the following equation [43]:

1 $$ {D}_n=\left(\frac{R_k}{R_{\mathrm{w}}}\right)\ {L}^2\frac{1}{\tau_{\mathrm{eff}}} $$

**Table 3:** EIS results of the TiO_2_ NS- and P-25-based QDSSCs

Electrode	*τ*_eff_ (ms)	*R*_*w*_ (ohm)	*R*_*k*_ (ohm)	*D*_*n*_ (cm^2^/s)	*L*_*n*_ (μm)
TiO_2_ NSs	6.54	5.96	28.26	7.25 × 10^−4^	21.78
P-25	4.93	8.98	8.13	1.84 × 10^−4^	9.52

From the phase Bode plots, inset of Fig. 6a, we can get the characteristic peak frequency of the QDSSC, *f*_peak_, and the first-order reaction rate constant for electron loss, *k*_eff_ ≈ 2*πf*_max_. The *τ*_eff_ can then be estimated as follows:

2 $$ {\tau}_{\mathrm{eff}}\approx \frac{1}{k_{\mathrm{eff}}} $$

The TiO_2_ NS-based QDSSC has a lower characteristic peak frequency compared with the P-25-based QDSSC, indicating the electrons in the TiO_2_ NSs can diffuse further. The result reveals the employment of the nanosheet structure favors the electron transport and suppresses the charge recombination. The fitted smaller *R*_*w*_ and larger *R*_*k*_ for the TiO_2_ NS-based QDSSC also confirm the result. The smaller *R*_*w*_ for the TiO_2_ NS-based QDSSC indicates the connection network of the highly crystalline (001) facets offers a better-oriented electron pathway, which minimizes the grain interface effect and reduces the electron loss from TiO_2_ NSs to the FTO substrate. Likewise, the fitting result also shows that the TiO_2_ NS-based QDSSC has a larger *R*_*k*_ (28.26 Ω) than the P-25-based QDSSC (8.98 Ω). The larger *R*_*k*_ presents higher resistance for the electron recombination process, due to the higher surface coverage of QDs on the TiO_2_ NSs, resulting in more electrons surviving from the back reaction at the uncovered TiO_2_-NS/electrolyte interface. Previous reports using the ZnS passivation treatment technique on the P-25-based QDSSCs also showed similar results [40]. The corresponding electron diffusion length *L*_*n*_ of TiO_2_ NSs was estimated to be ~ 21 μm, which is two times longer than that of P-25. In addition, the *L*_*n*_ of TiO_2_ NSs is found much longer than the thickness of the photoanodes (21 μm vs. 10 μm), implying most of the photogenerated electrons can be collected without recombination. The high electron collection efficiency in the TiO_2_ NS film was manifested by the high IPCE value.

## Conclusions

2D anatase TiO_2_ NSs with high (001)-exposed facets have been prepared by a facile hydrothermal process and used as the photoanodes for the CdS/CdSe co-sensitized solar cells (Fig. 5). The TEM study and UV-VIS absorption spectra show highly crystalline TiO_2_ NSs with over 70% of (001) facets. Both the TiO_2_ NS- and P-25-based QDSSCs are characterized in terms of the photovoltaic performance as well as the dynamics of electron transport and recombination. The TiO_2_ NS-based QDSSC can perform an overall energy conversion efficiency of 4.42%, which corresponds to 54% enhancement in comparison with the P-25-based cell (2.86%) under similar fabrication conditions. Furthermore, the IPCE value of over 70% can be achieved in the wavelength range of 450–600 nm for the TiO_2_ NS-based QDSSC, attributed by the higher light harvesting and electron collection efficiency of the TiO_2_ NS photoanode. The EIS analysis also confirms the dominant (001) facets of TiO_2_ NSs can dramatically improve the power conversion efficiency of the TiO_2_-based CdS/CdSe-sensitized QDSSCs system. This finding reveals the possibility of exploiting the (001)-oriented TiO_2_ NSs in colloidal QDSSC application since the QDs can be anchored probably on the TiO_2_ NSs without the need of extra linkers (which are electron transfer barriers between the QDs and TiO_2_ in most cases). In addition, the utilization of TiO_2_ NSs in this work has shown the following benefits: stable, mass production, cheap, etc., since the fabrication process is not complicated and does not need expensive additives.

## Additional file

Additional file 1: Calculation of the percentage of the exposed (001) facets in anatase TiO_2_ NSs and NPs. (DOCX 59 kb)

## Abbreviations

CBD: Chemical bath deposition
DSSCs: Dye-sensitized solar cells
EIS: Electrochemical impedance spectroscopy
FE-SEM: Field emission scanning microscopy
FTO: Fluorine-doped tin oxide
ICP-MS: Inductively coupled plasma mass spectrometer
IPCE: Incident photon converted to current efficiency
NPs: Nanoparticles
NSs: Nanosheets
QDs: Quantum dots
QDSSCs: Quantum dot-sensitized solar cells
SILAR: Successive ion layer absorption and reaction
TEM: Transmission electron microscopy
XRD: X-ray diffraction
