# Supplementary material for: Performance Enhancement of CdS/CdSe Quantum Dot-Sensitized Solar Cells with (001)-Oriented Anatase TiO2 Nanosheets Photoanode
Source: Nanoscale Res Lett. 2019 Jan 11;14:18. doi: 10.1186/s11671-018-2842-5 (PMC6329687; doi:10.1186/s11671-018-2842-5)
Supplement: Supplementary file 1 — Calculation of the percentage of the exposed (001) facets in anatase TiO2 NSs and NPs. (DOCX 59 kb) [file 11671_2018_2842_MOESM1_ESM.docx]

**Additional file 1**

**Performance enhancement of CdS/CdSe quantum dot sensitized solar cells with (001)-oriented anatase TiO_2_ nanosheets photoanode**

(Manuscript Number: NARL-D-18-00600)

- *Calculation of the percentage of the exposed (001) facets in anatase TiO_2_ NSs and NPs:*

From SEM, HRTEM and XRD analyses, we can construct the structures of anatase TiO_2_ NS and NP, as shown below.

(001)

(101)

Width: W

Length: L

W’

L'

θ

t

Width: W

(001)

(101)

Length: L

W’

L'

θ

t

A- TiO_2_ NS

A- TiO_2_ NP

For anatase TiO_2_ NSs, the angle $\theta$between the (001) and (101) facets has been found to be 68.3^o^ [Ref. 1]. The percentage of the exposed (001) facets can then be estimated using the following equation [Ref. 2]:

$$\frac{W'*L'*2}{W^{'}*L^{'}*2+\frac{L^{2}-{L^{'}}^{2}}{4\cos\theta}*4+\frac{W^{2}-{W^{'}}^{2}}{4\cos\theta}*4}$$

where L, W, and t are respectively the length, width, and thickness of TiO_2_ NS, and L = L’+$t/\tan\theta$ and W = W’ +$t/\tan\theta$. We randomly chose various NSs and obtain the percentage of the exposed (001) facets approximately equal to 70%.

By the same way, the percentage of exposed (001) facets in anatase P-25 NPs was found roughly below 10%. However, it is worth to note that only about 80 % of P-25 NPs are anatase, i.e., about 20% are rutile. Therefore, for the P-25 NPs, the percentage of exposed (001) facets is less than 10%, with over 90% dominated by the (101), (110), etc. facets.
